# Reply to Hagen and Thornhill

**DOI:** 10.1093/emph/eow033

**Published:** 2017-01-10

**Authors:** Sarah Myers, Oskar Burger, Sarah E Johns

**Affiliations:** School of Anthropology and Conservation, Marlowe Building, University of Kent, Canterbury, Kent CT2 7NR, UK

We thank Hagen and Thornhill for their thoughtful comments on our paper [1]. We appreciate, based on the explanation provided in their commentary, that model we tested differed somewhat from their psychic pain hypothesis (PPH). Furthermore, we are pleased that our article has sparked debate and has afforded them the opportunity to further clarify their ideas for the purposes of future analysis. It is important that evolutionary hypotheses are testable and that empirical evidence is brought to bear on them, particularly when they have the potential to positively influence public health as evolutionary approaches to mental health do. For instance, at a time when population ageing is a major source of financial strain in many industrialized societies and governments are seeking to encourage higher fertility [2], our findings highlight a potential new source of financial incentive to invest in postnatal depression (PND) screening and preventative measures.

It is important to clarify when designing our study we did not have the authors work explicitly in mind. Rather, our starting point was the medical literature documenting the costly nature of experiencing depression. Our primary interest was in understanding these costs from a life history perspective. From this starting point we set out to explore what impact PND had on fertility and parity progression, following a call from demographers to address this gap in the literature [3]. Our research is the first to specifically investigate the consequences on PND on female reproductive decision making, adding to a growing literature on the importance of maternal wellbeing for female parity progression [4], irrespective of how the findings fit within a general evolutionary framework or specifically within that of the PPH.

The maternal circumstances variable which Hagen and Thornhill critique was created in response to reviewers’ feedback on our original work requesting moderation analysis to explore whether the detrimental impacts of PND on parity progression held across women of differing circumstances (which we found that they did). The measure is a composite of other measures which were collected for the purpose of controlling for factors known to influence fertility, rather than specifically as measures of maternal condition, and we readily admit this is not a ‘perfect’ measure (if such a thing exists). Future work should seek to improve upon this, perhaps by collecting data on more robust markers of socioeconomic status, health markers, partner relationship dynamics and extrinsic mortality risk.

Hagen and Thornhill propose a number of ways in which researchers may test their PPH in the future, which can only be welcomed. In their commentary, the authors make the uncontentious points that (i) signalling mechanisms may evolve to over-fire and (ii) various factors indicating maternal condition, and influencing both PND risk and fertility, are highly correlated between parity levels. Thus, it may not be surprising, as our results indicate, that women in positive circumstances sometimes have PND, and that repeat bouts of PND are correlated with reduced fertility. Another way of compiling evidence in support of the PPH was brought to our attention by an anonymous reviewer. They suggested our data could be used to look at parity progression in those women whose poor circumstances *do* improve between births, and thus might be expected to continue childbearing. In this group, PND should be predicted to have a positive effect on the parity progression of women whose poor circumstances ‘appropriately’ trigger the PND mechanism because they potentially will have successfully elicited extra resources via social subsidy (as predicted by the PPH). Whereas women whose PND mechanism did not trigger, on the other hand, would not have benefited from extra resources as a result of PND, and will thus be less likely to have more offspring compared to the PND experiencing women.

We briefly explore this possibility by testing the following hypotheses: to assess whether our maternal circumstances variable is sensitive enough to predict the likelihood of shifts in parity progression we test the hypothesis that (a) among women in poor circumstances at their first birth, improvement in circumstances between parities 1 and 2 will have a positive effect on the likelihood of progressing to parity 3, and (b) women who have poor circumstances at their first birth, but whose circumstances improved at their second birth, will be more likely to have a third birth if they had PND at their first birth compared to if they did not have PND at their first birth.(*a*) *Methods*. Women were first selected on the basis of their having a *maternal circumstances* score ≥ 2 at parity 1, indicating two or more poor category ratings (*n* = 154) (for more details see [1]). A binary logistic regression model was then run with parity progression from parity 2 acting as the dependent variable, and *whether maternal circumstances stayed the same/deteriorated or improved between parities 1 and 2* acting as a categorical predictor, while controlling for the demographic factors *age at second birth* and *year of mother’s birth*.

*Results* *.* There was a trend for women whose circumstances improved between her first and second births to have increased odds of having a third birth (OR 2.010, *P *= 0.057) (Table 1).

**Table 1.** Results of binary logistic regression models assessing hypotheses (a) and (b). Variable of interest shown in bold.

Variable		*b*	SE	Wald	df	*P*	Odds ratio	95% CI for odds ratio	
								Lower	Upper
*(a*) Does improvement in maternal circumstance increase the likelihood of parity progression									
**Did maternal circumstances improve?**	**Yes**	**0.698**	**0.367**	**3.614**	**1**	**0.057**	**2.010**	**0.979**	**4.129**
	**No (ref)**	−	−	−	−	−	−	−	−
Age at birth (years)		−0.131	0.039	11.296	1	0.001	0.877	0.813	0.947
Year of mother's birth		0.011	0.023	0.236	1	0.627	1.011	0.967	1.057
Constant		−18.503	44.183	0.175	1	0.675	0.000	–	–

(*b*) Does PND increase the likelihood of parity progression in women whose circumstances improve									
**PND**	**Yes**	−**1.190**	**0.546**	**4.752**	**1**	**0.029**	**0.304**	**0.104**	**0.887**
	**No (ref)**	−	−	−	−	−	−	−	−
Age at birth (years)		−0.164	0.057	8.374	1	0.004	0.849	0.760	0.949
Year of mother's birth		0.050	0.032	2.458	1	0.117	1.051	0.988	1.118
Constant		−91.885	61.519	2.231	1	0.135	0.000	–	–

Pseudo *R*^2^: (*a*) Cox and Snell 0.090, Nagelkerke 0.123; (*b*) Cox and Snell 0.154, Nagelkerke 0.207.

Although only approaching significance, we take this as indicating hypothesis *b* is at least worth exploring. (*b*) *Methods*. Next, from this sample, women were further selected on the basis of their having a positive score when their *maternal circumstances* score at parity 2 was subtracted from their score at parity 1, indicating their circumstances improved (*n* = 83). A binary logistic regression model was then run with parity progression from parity 2 acting as the dependent variable, and *PND incidence (EPDS) at parity 1* (for more details see [1]) acting as the predictor, while also controlling for the demographic factors *age at second birth* and *year of mother’s birth*.

*Results*. PND in association with a woman’s first birth reduced the odds of her having a third birth by 70% (OR 0.304, *P *= 0.029) (Table 1).

It has previously been noted that adaptationist explanations for depression suffer from a lack of identifiable beneficiaries [5]; had we found that PND increased the likelihood of parity progression, either here or in our original paper, then this would arguably have supported the case for PND being an adaptive mechanism to help mothers offset the costs of childrearing. We acknowledged in our original paper [1] that, as Hagen and Thornhill suggest, existing offspring may benefit in terms of investment from their mothers ceasing to reproduce. However, we feel given the detrimental impacts of depression on maternal health and infant development, our results are better interpreted as further evidence of the extremely costly nature of depression which indicates PND would be poorly designed as a signalling mechanism to inform mothers of their already impoverished state. The data presented in our original paper is the first to be published from a wider study investigating the consequences of PND on a range for fitness markers; we aim to bring further empirical evidence to this topic in the near future. We hope other researchers follow Hagen and Thornhill’s suggestions for collecting data specifically to test the PPH and look forward to the ensuing debate.

**Conflict of interest**: None declared.
